# Development of a Method for the Preparation of Ruthenium Indenylidene-Ether Olefin Metathesis Catalysts

**DOI:** 10.3390/molecules17055675

**Published:** 2012-05-11

**Authors:** Leonel R. Jimenez, Daniel R. Tolentino, Benjamin J. Gallon, Yann Schrodi

**Affiliations:** Department of Chemistry and Biochemistry, California State University, Northridge, 18111 Nordhoff Street, Northridge, CA 91330, USA

**Keywords:** olefin metathesis, ring-closing metathesis, ruthenium indenylidene, ruthenium alkylidene

## Abstract

The reactions between several derivatives of 1-(3,5-dimethoxyphenyl)-prop-2-yn-1-ol and different ruthenium starting materials [*i.e.*, RuCl_2_(PPh_3_)_3_ and RuCl_2_(p-cymene)(L), where L is tricyclohexylphosphine di-*t*-butylmethylphosphine, dicyclohexylphenylphosphine, triisobutylphosphine, triisopropylphosphine, or tri-*n*-propylphosphine] are described. Several of these reactions allow for the easy, *in-situ* and atom-economic preparation of olefin metathesis catalysts. Organic precursor 1-(3,5-dimethoxyphenyl)-1-phenyl-prop-2-yn-1-ol led to the formation of active ruthenium indenylidene-ether complexes, while 1-(3,5-dimethoxyphenyl)-prop-2-yn-1-ol and 1-(3,5-dimethoxyphenyl)-1-methyl-prop-2-yn-1-ol did not. It was also found that a bulky and strong σ-donor phosphine ligand was required to impart good catalytic activity to the new ruthenium complexes.

## 1. Introduction

Metal alkylidene complexes have been the focus of intense research in synthetic chemistry [1]. Most notable are the Schrock molybdenum and tungsten alkylidene [2] and the Grubbs ruthenium alkylidene complexes [3], which are excellent catalysts for olefin metathesis and have enabled an astonishingly broad spectrum of applications in organic and polymer synthesis [4]. Molybdenum alkylidene complexes find many important uses including in enantioselective metathesis reactions [5], while certain tungsten alkylidene systems were recently shown to be suitable catalysts for *Z*-selective metathesis [6]. Ruthenium alkylidene systems have been the most widely used olefin metathesis catalysts in industrial and academic laboratories, because they combine robustness, functional group tolerance, excellent activity, and good selectivity including enantioselectivity [5,7,8,9] and *Z*-selectivity [10].

**Figure 1:**
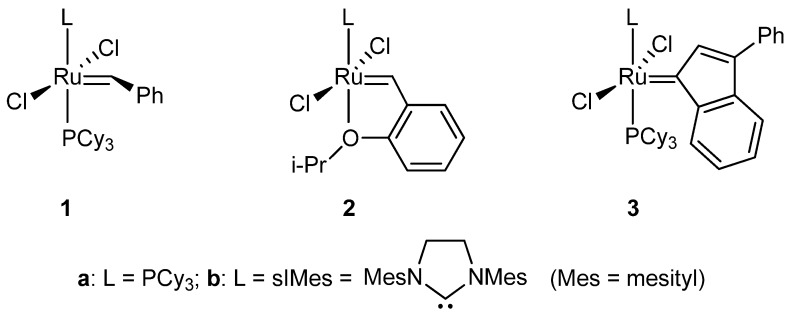
Ruthenium alkylidene olefin metathesis catalysts.

Many ruthenium-based olefin metathesis catalysts are commercially available including members of the ruthenium benzylidene (**1**) [11,12], benzylidene-ether (**2**) [13,14], and indenylidene (**3**) families (Figure 1) [15,16]. The preparations of these complexes are not straightforward, but involve several steps and require crystallization of the catalysts to remove phosphine byproducts. For example, the most practical method to produce the 1st generation Hoveyda catalyst **2a** consists of first preparing and isolating the 1st generation Grubbs complex **1a** in a two-step, one-pot process [11], and subsequently reacting **1a** with an alkoxystyrene molecule to give **2a** and one equivalent of tricyclohexylphosphine (PCy_3_) [13]. The end of the process requires isolating the catalyst **2a** away from the liberated phosphine. In addition to being cumbersome, this catalyst synthesis is not atom economic, wasting several equivalents of triphenylphosphine (PPh_3_) and one equivalent of the expensive PCy_3_ ligand. Therefore, we became motivated to develop a direct, *in-situ*, and atom-economic method for the synthesis of olefin metathesis catalysts.

The preparation of the ruthenium indenylidene complex **3a** is comparable with that of **1a** in some respects, because it also involves a two-step, one-pot process followed by isolation of the catalyst away from the liberated PPh_3_. On the other hand, making **3a** seems more attractive because it avoids the use of diazo compounds and does not require cooling of the reaction mixtures. However, an analysis of the history of **3a**’s discovery and additional studies reveals that its formation is not as straightforward as it initially appears. Indeed, the reaction between RuCl_2_(PPh_3_)_3_ and 1,1-diphenylprop-2-yn-1-ol (HC≡CCPh_2_OH) was first studied by Hill and reported to yield allenylidene complex RuCl_2_(PPh_3_)_2_(=C=C=CPh_2_) [17]. Conversely, Nolan [18] and Fürstner [19] described that the same reaction under very similar conditions (reflux in THF for 2 h) yields the indenylidene complex RuCl_2_(PPh_3_)_2_(PhInd) (where PhInd is a 3-phenyl-1-indenylidene fragment). Schanz found the preparation of RuCl_2_(PPh_3_)_2_(PhInd) under the aforementioned conditions to be unreliable and showed that it affords the dinuclear species (PPh_3_)_2_ClRu(µ-Cl)_3_Ru(PPh_3_)_2_(=C=C=CPh_2_) [20]. It is also interesting to note that [RuCl_2_(*p*-cymene)]_2_ reacts with HC≡CCPh_2_OH in the presence of two equivalents of PCy_3_ under similar conditions as above to give RuCl_2_(PCy_3_)_2_(=C=C=CPh_2_), which does not rearrange to RuCl_2_(PCy_3_)_2_(PhInd) [18]. In contrast, the formation of RuCl_2_(PPh_3_)_2_(PhInd) from the reaction of RuCl_2_(PPh_3_)_3_ and HC≡CCPh_2_OH is thought to go through an allenylidene-to-indenylidene rearrangement involving an electrophilic aromatic substitution of one of the allenylidene’s phenyl groups [20]. A similar rearrangement was observed by Dixneuf in a cationic ruthenium allenylidene system [21].

Altogether, we were attracted by the use of propargyl alcohol derivatives to generate metal alkylidene complexes, but desired to design 1-phenylprop-2-yn-1-ol precursors that would favor the formation of metal indenylidene over metal allenylidene complexes. We reasoned that prop-2-yn-1-ol molecules containing an activated phenyl ring would be suitable precursors. We have previously communicated the convenient, atom-economic, and *in-situ* preparation of a new indenylidene-ether olefin metathesis catalyst using 1-(3,5-dimethoxyphenyl)-1-phenylprop-2-yn-1-ol [22]. Herein, we describe the path that led to the development of this synthesis as well as the reaction of other organic precursors with different ruthenium starting materials.

## 2. Results and Discussion

### 2.1. Synthesis of the 1-Phenylprop-2-yn-1-ol Derivatives

**Scheme 1:**
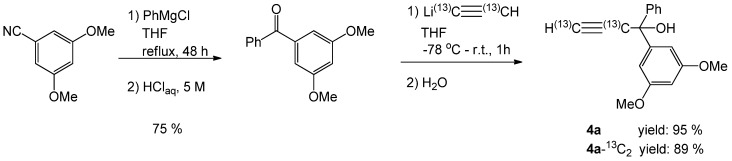
Synthesis of 1-(3,5-dimethoxyphenyl)-1-phenylprop-2-yn-1-ol.

Two organic precursors, 1-(3,5-dimethoxyphenyl)-1-phenylprop-2-yn-1-ol (**4a**) and its isotopologue (**4a**-^13^C_2_), were initially prepared (Scheme 1). 3,5-Dimethoxybenzophenone, obtained by reacting 3,5-dimethoxybenzonitrile with phenylmagnesium chloride, was treated with lithium acetylide (LiC≡CH and Li^13^C≡^13^CH) at −78 °C in THF to give the non-labeled **4a** and ^13^C-doubly-labeled **4a**-^13^C_2_ organic precursors, respectively. The ^1^H-NMR spectrum of **4a** in CDCl_3_ showed characteristic resonances for the alcohol group (singlet at 2.84 ppm) and the acetylenic proton (singlet at 2.93 ppm). The resonance for the protons of the methoxy groups appear as a singlet at 3.74 ppm. The ^13^C-NMR spectrum of **4a** features a total of twelve peaks including two singlets at 86.45 and 75.56 ppm for the acetylenic carbon atoms. In **4a**-^13^C_2_, the acetylenic proton is characterized by a doublet of doublets (^1^*J*_C-H_ = 250.4 Hz and ^2^*J*_C-H_ = 50.0 Hz) in the ^1^H-NMR spectrum and the acetylenic carbon atoms by two doublets (^1^*J*_C-C_ = 171.5 Hz) in the ^13^C-NMR spectrum.

**Scheme 2:**
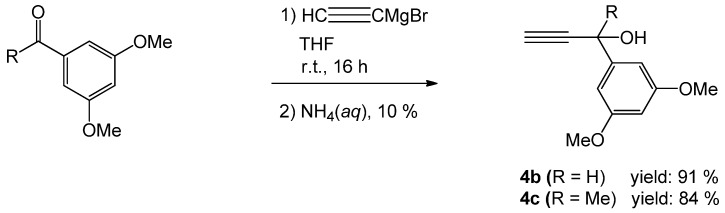
Synthesis of smaller derivatives of 1-phenylprop-2-yn-1-ol.

We were also interested in making organic precursors that were less bulky than **4a** (*vide infra*) and whose synthesis would not require the use of *n*-butyllithium. Thus, 1-(3,5-dimethoxyphenyl)-prop-2-yn-1-ol (**4b**) and 1-(3,5-dimethoxyphenyl)-1-methylprop-2-yn-1-ol (**4c**) were straightforwardly produced by reacting ethynylmagnesium bromide with 3,5-dimethoxybenzaldehyde and 3,5-dimethoxyacetophenone, respectively (Scheme 2). The ^1^H-NMR spectrum of **4b** in CDCl_3_ showed a singlet at 2.64 ppm for the acetylenic proton. The resonance for the protons of the methoxy groups appears as a singlet at 3.78 ppm, and that for the propargylic proton is found as a singlet at 5.37 ppm. The ^1^H-NMR spectrum of **4c** in CDCl_3_ is characterized by a singlet at 2.66 ppm for the acetylenic proton, a singlet at 1.76 ppm for the propargylic methyl group, and a singlet at 3.78 ppm for the methoxy substituents.

### 2.2. Reaction of 1-Phenylprop-2-yn-1-ol Derivatives with Different Ruthenium Starting Materials

**Scheme 3:**
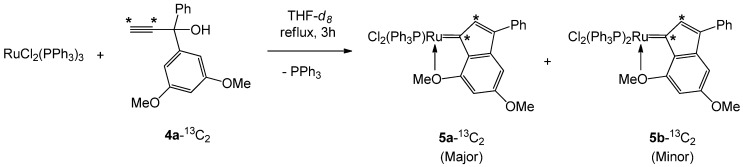
Reaction between RuCl_2_(PPh_3_)_3_ and **4a**-^13^C_2_.

Compound **4a** was reacted with RuCl_2_(PPh_3_)_3_ in refluxing THF-*d_8_*. ^31^P-NMR spectroscopy revealed that complete disappearance of RuCl_2_(PPh_3_)_3_ occurred after 3 h to give free PPh_3_ (singlet at −4.9 ppm) and two new species in a ~6:1 ratio (singlet at 53.4 ppm for the major species and singlet at 27.5 ppm for the minor species). The methoxy groups of both the major and minor species seemed to be inequivalent, according to ^1^H-NMR spectroscopy, suggesting that the products could be ruthenium indenylidene complexes. Nevertheless, a firmer elucidation of the nature of the organometallic products was hampered by the lack of information in the ^1^H-NMR spectrum and the low intensities of the low-field signals in the ^13^C-NMR spectrum. Thus, we began to explore the reaction of RuCl_2_(PPh_3_)_3_ with the ^13^C-doubly labeled organic precursor **4a**-^13^C_2_ in refluxing THF-*d_8_*. After 3 h of reaction, the ^13^C{^1^H} spectrum of the mixture features a doublet of doublets at 288.9 ppm (^1^*J*_Cα-Cβ_ = 49.6 Hz; ^2^*J*_Cα-P_ = 12.2 Hz) for the ^13^C_α_ nucleus of the major species, consistent with the C_α_ nucleus coupling to one ^31^P nucleus and to the ^13^C_β_ nucleus. Further down field, the spectrum exhibits a doublet of triplets at 301.1 ppm (^1^*J*_Cα-Cβ_ = 41.3 Hz; ^2^*J*_Cα-P_ = 14.2 Hz) for the ^13^C_α_ nucleus of the minor species, consistent with the C_α_ nucleus coupling to two ^31^P nuclei and to the ^13^C_β_ nucleus. The spectrum also exhibits a doublet of doublets at 143.8 ppm for the ^13^C_β_ nucleus of the major species and a doublet of triplets at 140.2 ppm for the ^13^C_β_ nucleus of the minor species, but lacks any resonance in the 250–220 ppm region. These data suggest that no appreciable amount of ruthenium allenylidene complex was formed and are consistent with the major species being a mono-phosphine ruthenium indenylidene complex **5a**-^13^C_2_ and the minor species a bis-phosphine ruthenium indenylidene **5b**-^13^C_2_ (Scheme 3) [17,18,19,20]. The ^31^P-NMR spectrum shows a doublet of doublets at 53.4 ppm for the major species and a doublet of doublets at 27.5 ppm for the minor species. The configuration of the ligands around the ruthenium center, including whether the chlorides adopt a *cis*- or *trans*-arrangement, is not known based on the above data.

**Scheme 4:**
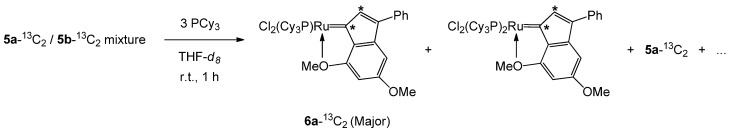
Phosphine exchange on complexes **5a**-^13^C_2_ and **5b**-^13^C_2_.

The mixture of **5a**-^13^C_2_ and **5b**-^13^C_2_ in THF-*d_8_* obtained above was treated with three equivalents of PCy_3_ per ruthenium atom at room temperature to give a mixture of organometallic species containing a new mono-phosphine ruthenium indenylidene complex **6a**-^13^C_2_ as a major component (Scheme 4). **6a**-^13^C_2_ is characterized by a doublet at 48.6 ppm (^2^*J*_Cα-P_ = 11.1 Hz) in the ^31^P-NMR spectrum, and in the ^13^C-NMR spectrum, by a doublet of doublets at 287.0 ppm (^1^*J*_Cα-Cβ_ = 49.0 Hz; ^2^*J*_Cα-P_ = 11.2 Hz) for the ^13^C_α_ nucleus and a doublet at 129.2 ppm (^1^*J*_Cα-Cβ_ = 49.0 Hz) for the ^13^C_β_ nucleus.

**Scheme 5:**
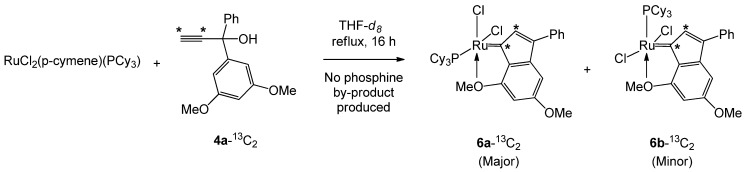
Reaction between RuCl_2_(p-cymene)(PCy_3_) and **4a**-^13^C_2_.

Complex **6a**-^13^C_2_ can also be generated as the major product by reacting RuCl_2_(p-cymene)(PCy_3_) with organic precursor **4a**-^13^C_2_ upon reflux in THF-*d_8_* for 16 h (Scheme 5) [22]. This reaction also produces a minor product **6b**-^13^C_2_, whose ^31^P-NMR spectrum features a doublet at 68.1 ppm (^2^*J*_Cα-P_ = 15.2 Hz) and whose ^13^C-NMR spectrum shows a doublet of doublets at 256.2.0 ppm (^1^*J*_Cα-Cβ_ = 47.8 Hz; ^2^*J*_Cα-P_ = 15.0 Hz) for the ^13^C_α_ nucleus and a doublet at 129.2 ppm (^1^*J*_Cα-Cβ_ = 48.9 Hz) for the ^13^C_β_ nucleus. A mixture of non-labeled **6a**/**6b** complexes was prepared on a gram scale by a similar procedure. However, efforts to isolate and purify the major and minor products **6a** and **6b** by silica gel chromatography and crystallization have been unsuccessful.

Bruneau and coworkers independently prepared a related complex by a similar approach utilizing 1,1-bis-(3,5-diisopropoxyphenyl)prop-2-yn-1-ol as an organic precursor [23]. The NMR spectroscopy data for their complex is very similar to those for our minor species (**6b**-^13^C_2_): The ^31^P-NMR spectrum shows a resonance at 68.2 ppm and the ^13^C-NMR spectrum shows resonances at 258.8 ppm (^2^*J*_Cα-P_ = 14.2 Hz) and 136.3 ppm for the ^13^C_α_ and ^13^C_β_ nuclei, respectively. Additionally, Bruneau and coworkers obtained a crystal structure of their complex showing it to be a mono-PCy_3_ ruthenium indenylidene-ether complex where the phosphine and ether ligands are *trans* to each other. We propose that **6a** and **6b** may be isomers, where the minor species **6b** possesses a structure similar to that of Bruneau’s complex (*trans*-phosphine-ether arrangement) and where the major complex **6a** adopts a *cis*-phosphine-ether arrangement as the more stable isomer, due to the reduced steric of the methoxy group in **6** compared to the bulkier isopropoxy group in Bruneau’s complex. Similarly, a relatively unhindered ruthenium alkylidene-pyridine complex supported by a NHC ligand was shown to exist as a major isomer adopting a *cis*-NHC-pyridine arrangement and a minor isomer with a *trans*-NHC-pyridine configuration [24].

A solution of complexes **6a** and **6b**, prepared *in situ* using the non-labeled organic precursor **4a** following a similar procedure as that shown in scheme 5, can be used without additional treatment to catalyze ring-closing metatheses (RCM) to produce 5-, 6-, and 7-membered disubstituted cycloalkenes with activities similar to those of the 1st generation Hoveyda catalyst **2a** under standard conditions [22,25]. In order to explore the influence of the phosphine ligand on the activity of these *in-situ* generated ruthenium indenylidene-ether complexes, other RuCl_2_(p-cymene)(L) starting materials, where L is di-*t*-butylmethylphosphine [P(tBu)_2_Me], dicyclohexylphenylphosphine [P(Cy)_2_Ph], triisobutylphosphine [P(iBu)_3_], triisopropylphosphine [P(iPr)_3_], or tri-*n*-propylphosphine [P(nPr)_3_], were reacted with organic precursor **4a** in refluxing THF-*d_8_* for 16 h. ^31^P-NMR spectroscopy showed that each one of these reactions affords new organometallic species (Table 1). The activity of these complexes in ring-closing metathesis is described in Section 2.3.

We also explored the possibility of using 1-(3,5-dimethoxyphenyl)-prop-2-yn-1-ol (**4b**) and 1-(3,5-dimethoxyphenyl)-1-methylprop-2-yn-1-ol (**4c**) as organic precursors for the preparation of ruthenium indenylidene-ether complexes. This study was motived by two main factors. First, compounds **4b** and **4c** are easier to prepare than **4a** (see above). Second, we hypothesized that less hindered indenylidene fragments may lead to faster-initiating catalysts based on a comparison of the RCM activity of our and Bruneau’s catalysts (Section 2.3). According to ^1^H- and ^31^P-NMR spectroscopy, RuCl_2_(p-cymene)(PCy_3_) does not react with either **4b** or **4c** in THF-*d_8_* at room temperature after 18 h. Furthermore, heating the respective solutions at 40 °C for 18 h yields much starting materials and several unidentified new species. The reaction of RuCl_2_(p-cymene)(PCy_3_) with **4b** or **4c** in refluxing THF-*d_8_* for 18 h affords complicated mixtures of products. Similarly, refluxing a solution of RuCl_2_(PPh_3_)_3_ and **4c** for 3 h produces multiple species. On the other hand, RuCl_2_(PPh_3_)_3_ reacts quite cleanly with **4b** to generate a new species characterized by four doublets of equal intensity at 50.4 ppm (^2^*J*_P-P_ = 37.7 Hz), 47.1 ppm (^2^*J*_P-P_ = 38.3 Hz), 40.9 ppm (^2^*J*_P-P_ = 23.8 Hz), and 39.0 ppm (^2^*J*_P-P_ = 24.0 Hz) in the ^31^P-NMR spectrum. This ^31^P-NMR signature is very similar to that of asymmetric dinuclear vinylidene complexes (P-P)ClRu(µ-Cl)_3_Ru(P-P)(=C=CHR) [26], and almost identical to that of the asymmetric bimetallic allenylidene compound (PPh_3_)_2_ClRu(µ-Cl)_3_Ru(PPh_3_)_2_(=C=C=CPh_2_) [17,20]. The ^1^H-NMR spectrum shows equivalent methoxy groups, but does not exhibit any signals corresponding to a Ru=C=CHC(OH)Ph fragment [27], leading us to believe that (PPh_3_)_2_ClRu(µ-Cl)_3_Ru(PPh_3_)_2_(=C=C=CHAr) (where Ar = 3,5-dimethoxyphenyl) may have been formed. In any case, the reaction of RuCl_2_(PPh_3_)_3_ and **4b** does not form an indenylidene complex. Altogether, these results suggest that the derivatives of 1-phenylprop-2-yn-1-ol may need to bear two electron-withdrawing groups (e.g., aryl groups) in the propargylic position to be suitable precursors for the clean and efficient formation of ruthenium indenylidene complexes.

### 2.3. RCM Activity of the Ruthenium Indenylidene-Ether Complexes

**Figure 2:**
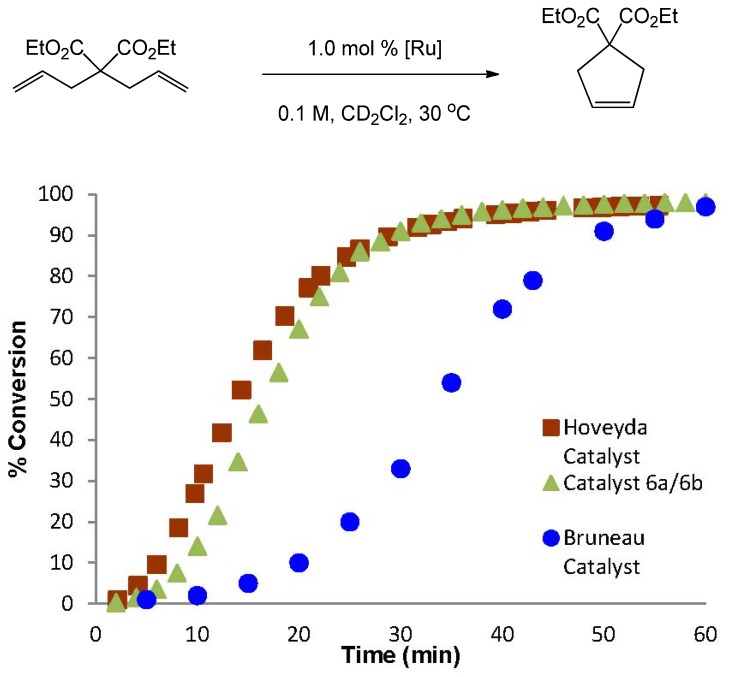
Activity comparison for catalysts **2a**, **6a**/**6b**, and Bruneau’s catalyst.

As mentioned above, a solution of **6a**/**6b** generated *in situ* efficiently promotes the formation of 5-, 6-, and 7-membered disubstituted cycloalkenes by RCM. Although the activity of our system is very similar to that of the 1st generation Hoveyda catalyst **2a** in the RCM of diethyl diallylmalonate (DEDAM) under standard conditions [22,25], it is interesting to note that Bruneau’s catalyst exhibits a longer initiation period (Figure 2) [23]. Indeed, at the 30 min time point, Bruneau’s catalyst has converted about 33% of the substrate to product, while our catalyst has reached greater than 90% conversion. A possible explanation for this is that the bulkier indenylidene ligand of Bruneau’s catalyst hinders the rotation around the Ru=C bond of the ruthenium indenylidene fragment, a rotation that may be necessary for the formation of the rotamer that initiates the olefin metathesis reaction. A similar rotation takes place in the Hoveyda catalysts, [28,29] whose activation is thought to involve dissociative and associative interchange pathways [30].

The activity of the solutions generated by the reaction of RuCl_2_(p-cymene)(L) and organic precursor **4a** (Table 1) was compared to that of the solution of **6a**/**6b** (L = PCy_3_) in the RCM of DEDAM. The catalytic system **6a**/**6b** bearing the PCy_3_ ligand is the most active, reaching greater than 97% conversion within 30 min at 1.0 mol % catalyst loading (Table 2; entry 1). The catalysts supported by P(tBu)_2_Me or P(iPr)_3_ ligands are less effective than **6a**/**6b** (Table 2; compare entries 2 and 6 to entry 1), but are still able to achieve greater than 90% conversion within 60 min at 2.0 mol % catalyst loadings (Table 2; entries 3 and 7). Conversely, the complexes containing the P(Cy)_2_Ph, P(iBu)_3_ or P(nPr)_3_ ligands show very low activity in the RCM of DEDAM under the tested conditions (Table 2; entries 4, 5 and 8).

These results follow the ligand effects observed by Grubbs and coworkers, namely that larger and more electron-donating phosphine ligands lead to more active catalysts [31]. These trends are easily rationalized for the 1st generation Grubbs systems which enter the catalytic cycle via a dissociative substitution of a phosphine with an olefin [32]. Thus, the ligand dissociation is favored by the large steric hindrance and the strong *trans* influence of ligands such as PCy_3_, producing more active species. The explanation for the trends within the ruthenium indenylidene-ether set of catalysts is less intuitive. Nevertheless, it seems reasonable that phosphine ligands with stronger electron-donating abilities may turn over faster by accelerating the olefin binding step [33] or by stabilizing the metallacyclobutane intermediate and metathesis transition states [34], as it was originally proposed in the case of *N*-heterocyclic carbene (NHC) *versus* phosphine ligands [35]. It is also conceivable that bulkier phosphine ligands are required to influence the Ru=C bond rotation favoring the formation of the active rotamer and driving the reaction toward the less sterically hindered intermediates and transition states [33,34].

## 3. Experimental

### 3.1. General

NMR spectra were recorded on a Bruker 400 MHz NMR spectrometer running Xwin-NMR software. Chemical shifts are reported in parts per million (ppm) downfield from tetramethylsilane (TMS) with reference to internal solvent for ^1^H-NMR and ^13^C-NMR spectra. Chemical shifts are reported in parts per million (ppm) downfield from H_3_PO_4_ for ^31^P-NMR spectra. All glassware was oven dried. Unless noted otherwise, all reactions were conducted under an atmosphere of argon (in an argon-filled glove box or under argon using Schlenk techniques). All organic solvents were dried by passage through solvent purification columns containing activated molecular sieves. All other commercial chemicals were used as obtained. Organic precursors **4a** and **4a**-^13^C_2_ [22], and RuCl_2_(p-cymene)(L) [36] [where L = PCy_3_, P(tBu)_2_Me, PCy_2_Ph, P(iBu)_3_, P(iPr)_3_, and P(nPr)_3_] were prepared according to literature procedures.

### 3.2. Preparation of the 1-Phenylprop-2-yn-1-ol Derivatives ***4b*** and ***4c***

#### 3.2.1. Preparation of 1-(3,5-Dimethoxyphenyl)-prop-2-yn-1-ol (**4b**)

A dry 100 mL reaction flask equipped with a stir bar was charged with 3,5-dimethoxybenzaldehyde (1.0 g, 6.0 mmol) and anhydrous THF (15 mL) inside the glove box, capped with a septum, and taken out of the glove box. The mixture was placed in a 0 °C ice bath. A 0.5 M solution of ethynylmagnesium bromide in THF (20 mL, 10 mmol) was added dropwise under stirring. The reaction mixture was allowed to warm up to room temperature and stirred for 16 h. A 10% aqueous solution of NH_4_Cl (40 mL) was added and the mixture stirred for 30 min. The product was extracted with ether (3 × 30 mL) and the combined organic layers were washed with water (50 mL) and brine (50 mL) before being dried with anhydrous magnesium sulfate. The filtrate was dried *in vacuo* to afford 1-(3,5-dimethoxyphenyl)-prop-2-yn-1-ol as an orange oil in 91% yield. ^1^H-NMR (CDCl_3_): δ 6.70 (s, 2H), 6.41 (s, 1H), 5.37 (s, 1H), 3.78 (s, 6H), 2.64 (s, 1H), proton from OH group not observed. ^13^C{^1^H} NMR (CDCl_3_): δ 160.96, 142.44, 104.55, 100.57, 83.42, 74.71, 64.35, 55.41.

#### 3.2.2. Preparation of 1-(3,5-Dimethoxyphenyl)-1-methylprop-2-yn-1-ol (**4c**)

Powdered potassium carbonate (27.3 g, 197.5 mmol) was added to a suspension of 3,5-dihydroxy-acetophenone (5.0 g, 32.9 mmol) in acetone (50 mL) and the mixture was stirred vigorously for 20 min. Methyl iodide (8.4 mL, 134.9 mmol) was added dropwise and the reaction mixture was refluxed for 16 h. The mixture was filtered and the solid washed with acetone. Water (100 mL) was added to the filtrate, and the product was extracted with ether (3 × 100 mL). The combined organic layers were washed with water (200 mL) before being dried with anhydrous magnesium sulfate. The filtrate was dried *in vacuo* to afford 3,5-dimethoxyacetophenone as a dark red oil in 93% yield. ^1^H-NMR (CDCl_3_): δ 7.09 (s, 2H), 6.65 (s, 1H), 3.82 (s, 6H), 2.57 (s, 3H). ^13^C-NMR (CDCl_3_): δ 197.76, 160.88, 139.10, 106.18, 105.34, 55.59, 26.72. A dry 100 mL reaction flask equipped with stir bar was charged with 3,5-dimethoxyacetophenone (0.5 g, 2.8 mmol) and anhydrous THF (10 mL) inside the glove box, capped with a septum, and taken out of the glove box. The mixture was placed in a 0 °C ice bath. A 0.5 M solution of ethynylmagnesium bromide in THF (9 mL, 4.5 mmol) was added dropwise under stirring. The reaction mixture was allowed to warm up to room temperature and stirred for 16 h. A 10% aqueous solution of NH_4_Cl (20 mL) was added and the mixture stirred for 30 min. The product was extracted with ether (3 × 30 mL) and the combined organic layers were washed with water (50 mL) and brine (100 mL) before being dried with anhydrous magnesium sulfate. The filtrate was dried *in vacuo* to afford 1-(3,5-dimethoxyphenyl)-1-methylprop-2-yn-1-ol as a brown oil in 84% yield. ^1^H-NMR (CDCl_3_): δ 6.82 (s, 2H), 6.39 (s, 1H), 3.80 (s, 6H), 2.66 (s, 1H), 1.76 (s, 3H), proton from OH group not observed. ^13^C{^1^H} NMR (CDCl_3_): δ 160.71, 147.65, 103.31, 99.66, 87.16, 72.96, 69.83, 55.29, 33.01.

### 3.3. Reaction between 1-Phenylprop-2-yn-1-ol Derivatives and Different Ruthenium Starting Materials

#### 3.3.1. NMR Study of the Reaction between RuCl_2_(PPh_3_)_3_ and 1-(3,5-dimethoxyphenyl)-1-phenylprop-2-yn-1-ol (**4a**)

1-(3,5-Dimethoxyphenyl)-1-phenylprop-2-yn-1-ol, **4a**, (12 mg, 0.0375 mmol, 1.2 equiv) was weighed in a 2 mL vial and RuCl_2_(PPh_3_)_3_ (30 mg, 0.03128 mmol, 1.0 equiv) was weighed in a separate 2 mL vial. The organic precursor **4a** was dissolved with THF-*d_8_* (0.5 mL) and this solution of **4a** transferred to the vial containing the RuCl_2_(PPh_3_)_3_ starting material. The mixture was then transferred to a J-Young tube, which was capped, brought out of the glove box and placed for 3 h in an oil bath set at 70 °C. The reaction mixture was analyzed by ^31^P-NMR. ^31^P-NMR (161 MHz, THF-*d_8_*): δ 53.4 (s; major species), 27.5 (s; minor species), −4.9 (s, PPh_3_).

#### 3.3.2. NMR study of the Reaction between RuCl_2_(PPh_3_)_3_ and 1-(3,5-Dimethoxyphenyl)-1-Phenylprop-2-yn-1-ol (**4a**-^13^C_2_)

The reaction between RuCl_2_(PPh_3_)_3_ and **4a**-^13^C_2_ was set up following the same procedure used for the reaction between RuCl_2_(PPh_3_)_3_ and **4a** (see sub-section 3.3.1.). The reaction mixture was analyzed by ^13^C and ^31^P-NMR. ^13^C{^1^H} NMR (100 MHz, THF-*d_8_*): δ 301.1 (dt, ^1^*J*_Cα-Cβ_ = 41.3 Hz; ^2^*J*_Cα-P_ = 14.2 Hz; C_α_ of minor species), 288.9 (dd, ^1^*J*_Cα-Cβ_ = 49.6 Hz; ^2^*J*_Cα-P_ = 12.2 Hz; C_α_ of major species), 143.8 (dd, ^1^*J*_Cα-Cβ_ = 49.4 Hz; ^3^*J*_Cβ-P_ = 3.7 Hz; C_β_ of major species), 140.2 (dt, ^1^*J*_Cα-Cβ_ = 41.2 Hz; ^3^*J*_Cβ-P_ = 5.3 Hz; C_β_ of minor species). ^31^P-NMR (161 MHz, THF-*d_8_*): δ 53.4 (dd, ^2^*J*_Cα-P_ = 12.0 Hz; ^3^*J*_Cβ-P_ = 3.1 Hz; major species), 27.5 (dd, ^2^*J*_Cα-P_ = 13.6 Hz; ^3^*J*_Cβ-P_ = 5.5 Hz; minor species), −4.9 (s, PPh_3_).

#### 3.3.3. Gram-Scale Preparation of the **6a**/**6b** Mixture

A 100 mL Schlenk tube equipped with a stir bar was charged with 1-(3,5-dimethoxyphenyl)-1-phenylprop-2-yn-1-ol, **4a**, (730 mg, 2.72 mmol, 1.1 equiv), RuCl_2_(p-cymene)(PCy_3_) (1.45 g, 2.47 mmol), and THF (25 mL). The Schlenk tube was sealed, brought out of the glove box, and placed under stirring for 16 h in an oil bath set at 70 °C. The volatiles were removed under vacuum. The brown residue was then dissolved with toluene (5 mL) in air and the toluene solution was slowly added to pentane (200 mL) in a 500 mL Erlenmeyer flask under vigorous stirring. The brown precipitate was collected by filtration and dried under vacuum overnight to yield 1.11 g of brown crystalline material (65% yield). ^31^P-NMR (161 MHz, THF-*d_8_*): δ 48.6 (s; major species), 68.1 (s; minor species).

#### 3.3.4. General Procedure for the Reaction between RuCl_2_(p-cymene)(L) and Organic Precursors **4a**

A 2 mL vial was charged with RuCl_2_(p-cymene)(L) (50 mg), the organic precursor **4a** (1.1 equiv), and THF-*d_8_* (0.5 mL). The mixture was then transferred to a J-Young tube, which was capped, brought out of the glove box, and placed in an oil bath set at 70 °C for 16 h. ^31^P-NMR spectra were recorded (see Table 1 in Results and Discussion).

#### 3.3.5. General Procedure for the Reaction between RuCl_2_(p-cymene)(PCy_3_) and Organic Precursors **4b** and **4c**

A 2 mL vial was charged with RuCl_2_(p-cymene)(PCy_3_) (30 mg, 0.051 mmol, 1.0 equiv), the organic precursor **4b** or **4c** (0.097 mmol, 1.9 equiv), and THF-*d_8_* (0.5 mL). The mixture was then transferred to a J-Young tube, which was capped and brought out of the glove box. The reactions were monitored at room temperature, 40 °C and 70 °C by ^1^H-NMR and ^31^P-NMR spectroscopy (see Results and Discussion).

#### 3.3.6. General Procedure for the Reaction between RuCl_2_(PPh_3_)_3_ and Organic Precursors **4b** and **4c**

RuCl_2_(PPh_3_)_3_ (30 mg, 0.031 mmol, 1.0 equiv) was weighed in a 2 mL vial and the organic precursor **4b** or **4c** (0.065 mmol, 2.1 equiv) was weighed in a separate 2 mL vial. The organic precursor was dissolved with THF-*d_8_* (0.5 mL) and this solution was transferred to the vial containing the RuCl_2_(PPh_3_)_3_ starting material. The mixture was then transferred to a J-Young tube, which was capped, brought out of the glove box, and placed for 3 h in an oil bath set at 70 °C. The reactions were analyzed by ^1^H-NMR and ^31^P-NMR spectroscopy (see Results and Discussion).

### 3.4. General Procedure for the RCM of Diethyl Diallylmalonate (DEDAM)

A 0.1 M stock solution of DEDAM was prepared in the glove box by dissolving DEDAM (60 mg, 0.25 mmol) in 2.44 mL of CD_2_Cl_2_. A portion of this 0.1 M DEDAM solution (0.5 mL, 50 µmol) was transferred to a NMR tube equipped with a screw-cap septum top. Separately, a 4 mL conical vial was charged with RuCl_2_(p-cymene)(L) (0.085 mmol) and **4a** (25 mg, 0.094 mmol, 1.1 equiv). The vial was then filled with THF to a 1.0 mL mark (calibrated) before dropping a spin vane in the solution. The vial was sealed, brought out of the glove box, and placed under stirring for 16 h in an oil bath set at 70 °C. A portion of this solution (6.0 µL for 1.0 mol %, and 12 µL for 2.0 mol % ruthenium loading) was added to the 0.1 M solution of DEDAM in CD_2_Cl_2_ (0.5 mL, 50 µmol) by injecting it with through the septum using a syringe outside the glove box. The NMR tube was then placed in an oil bath regulated at 40 °C and the reaction mixture was analyzed by ^1^H NMR spectroscopy after a period of time (30 and 60 min). The extent of conversion of the RCM reaction was determined by comparing the ratio of the integrals of the methylene protons in the substrate (dt, 2.61 ppm) with those in the product (s, 2.98 ppm).

## 4. Conclusions

Reactions between several derivatives of 1-(3,5-dimethoxyphenyl)-prop-2-yn-1-ol and different ruthenium starting materials (*i.e.*, RuCl_2_(PPh_3_)_3_ and RuCl_2_(p-cymene)(L), where L is tricyclohexylphosphine, di-*t*-butylmethylphosphine, dicyclohexylphenylphosphine, triisobutylphosphine, triisopropylphosphine, or tri-*n*-propylphosphine) have been explored and have led to the development of a straightforward method for the preparation of new ruthenium indenylidene-ether ring-closing metathesis catalysts. The method involves the reaction between RuCl_2_(p-cymene)(PCy_3_) and 1-(3,5-dimethoxyphenyl)-1-phenylprop-2-yn-1-ol in refluxing THF and possesses many advantages. First, it is a one-step process from the starting materials. Second, it does not require the use of difficult-to-handle diazo compounds and does not need to be conducted at low temperature. Third, it consumes only one equivalent of the expensive PCy_3_ ligand and is altogether very atom-economic. Fourth, it does not produce any inhibiting byproducts, allowing the catalyst solution to be used without further treatment. The resulting catalyst promotes the formation of 5-, 6-, and 7-membered disubstituted cycloalkenes with activities comparable to that of the commercial 1st generation Hoveyda catalyst under standard conditions.

Additionally, it was shown that the use of 1-(3,5-dimethoxyphenyl)-prop-2-yn-1-ol and 1-(3,5-dimethoxyphenyl)-1-methyl-prop-2-yn-1-ol did not lead to the formation of ruthenium indenylidene complexes, indicating that the 1-phenylprop-2-yn-1-ol derivatives may need to bear two aryl groups in the propargylic position to be suitable precursors for these types of reactions.

Finally, a study of the effects of the phosphine ligand on the ring-closing metathesis activity of these new ruthenium complexes revealed that larger and more electron-donating phosphine ligands lead to more efficient catalysts.

## Figures and Tables

**Table 1:** ^31^P-NMR shifts for RuCl_2_(p-cymene)(L) and for the major product of the reaction between RuCl_2_(p-cymene)(L) and **4a**. ^a^

**Ligand (L)**	**RuCl_2_(p-cymene)(L)** δ (ppm)	**Major Product** δ (ppm)
P(tBu)_2_Me	43.8	61.1
P(Cy)_2_Ph	19.5	50.0
P(iBu)_3_	22.9	44.2
P(iPr)_3_	35.7	58.1
P(nPr)_3_	16.1	40.8

^a^ THF-*d_8_*; reflux; 16 h.

**Table 2:**
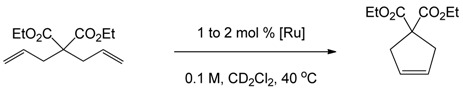
RCM of DEDAM with solutions prepared by reaction of RuCl_2_(p-cymene)(L) and **4a**. ^a^

**Entry**	**Ligand (L)**	**Ru loading** (mol %)	**Time** (min)	**Conversion** (%) *^b^*
1	PCy_3_	1.0	30	> 97
2	P(tBu)_2_Me	1.0	60	63
3	P(tBu)_2_Me	2.0	60	92
4	P(Cy)_2_Ph	2.0	60	> 3
5	P(iBu)_3_	2.0	60	4
6	P(iPr)_3_	1.0	60	68
7	P(iPr)_3_	2.0	60	95
8	P(nPr)_3_	2.0	60	> 3

*^a^* Solutions of RuCl_2_(p-cymene)(L) and **4a** in THF were refluxed for 16 h; *^b^* Determined by ^1^H-NMR analysis of the crude reaction mixture; >97% is indicated when no substrate was detected and <3% when no product was detected.
